# Organelle Crosstalk: Mitochondria and Endoplasmic Reticulum in Macular Degeneration

**DOI:** 10.26502/fjor.2644-002400117

**Published:** 2026-09-16

**Authors:** Iffat Alam, Ayeshah Hussain, Devendra K. Agrawal

**Affiliations:** Department of Translational Research, College of Osteopathic Medicine of the Pacific, Western University of Health Sciences, Pomona, California 91766 USA

**Keywords:** Age-Related Macular Degeneration (AMD), Epithelium, Endoplasmic Reticulum, Macular Degeneration, Mitochondria, Mitochondrial Associated Membranes (MAMs), Mitochondrial Dysfunction, Organelle Crosstalk, Oxidative Stress, Retinal Pigment Epithelium (RPE)

## Abstract

Age-related macular degeneration (AMD) is a leading cause of irreversible vision loss characterized by progressive retinal pigment epithelium (RPE) dysfunction, mitochondrial damage, oxidative stress, and chronic inflammation. While mitochondrial dysfunction is a known contributor to AMD, the contribution of the endoplasmic reticulum (ER) has not been fully explored. Mitochondrial associated membranes (MAMs) are regions of the mitochondria which are in direct contact with the ER and are critical areas for regulating essential cellular functions including calcium signaling, mitochondrial dynamics, and apoptosis. Dysfunction of these critical sites may lead to progressive retinal degeneration through the production of reactive oxygen species (ROS), calcium overload, and inflammasome activation in RPE cells. The study was conducted as a comprehensive critical review article on the subject. Findings are reported on the connection between the mitochondria and ER, specifically through MAM dysfunction, in macular degeneration. Overall, this critical review article discusses the pathophysiology of macular degeneration with the mechanistic insights into MAM deregulation and decoding the structural and functional breakdown of MAMs. Understanding the role of MAM-mediated signaling in AMD may identify potential therapeutic targets aimed at restoring mitochondrial resilience and retinal homeostasis.

## Introduction

Age-related macular degeneration (AMD) is a progressive, multifactorial ocular disease that is a leading cause for vision loss and blindness in individuals over 50, affecting ~196 million individuals worldwide [1]. The condition causes severe complications in individuals through macular degeneration which keeps peripheral vision somewhat intact in comparison to central vision loss, affecting basic visual tasks like reading and facial recognition. These symptoms are irreversible due to limited understanding of the pathogenesis and thus, lack of treatment options.

The irreversible vision loss in AMD is characterized by progressive retinal pigment epithelium (RPE) dysfunction, mitochondrial damage, oxidative stress, and chronic inflammation [2–4]. While the pathological mechanism is complex with genetic, age, and environmental contributions, studies have shown these factors converging to result in cellular damage and dysfunction, specifically of cells within the RPE. The RPE is the outermost layer of the retina essential for visual functions including a complex metabolic system that removes reactive oxygen species (ROS) and prevents oxidative damage. Damage to the structure leads to multiple retinopathies, including being tied to symptoms of aging and AMD [5]. Aging causes a decrease in RPE cell repair capacity, more mitochondrial dysfunction, and increased cell death. The accumulation of oxidative stress and ROS from dysfunctional mitochondria compounds and damages the RPE, thus leading to impaired function.

Mitochondria generate cellular energy and regulate cell life and death. Mitochondrial dysfunction may damage high-energy organs like the brain, heart, and muscles, and drives rare genetic disorders, common chronic illnesses [6–12]. While mitochondrial dysfunction is a known contributor to AMD, there has been an increase in attention upon the contribution of the endoplasmic reticulum (ER) [13–15]. The ER is essential for calcium homeostasis, protein folding, and lipid metabolism which has been linked to neurodegenerative diseases. However, the ER has not been fully explored in relation to the mitochondria and AMD.

Through investigation of association sites or connections between the mitochondria and ER, we may be able to discover new pathogenic pathways and targets for therapy. Mitochondrial associated membranes (MAMs) are regions of the mitochondria which are in direct contact with the ER and are critical areas for regulating essential cellular functions including calcium signaling, mitochondrial dynamics, and apoptosis. Dysfunction of these critical sites may lead to progressive retinal degeneration through the production of reactive oxygen species (ROS), calcium overload, and inflammasome activation in RPE cells (Figure 1).

The review article presents a comprehensive overview of the underlying pathogenesis of AMD and the current evidence of mitochondria and endoplasmic reticulum interactions and its contributions and connections to AMD.

### Mitochondrial Dysfunction in AMD

Mitochondrial dysfunction has been shown to be central to not only aging in general, but also the pathogenesis of AMD [16,17]. Specifically, the mitochondria within the RPE contribute heavily to vision loss through a cyclic damage pattern. The RPE is heavily dependent on mitochondrial efficiency and function for its highly metabolic activities involved in visual processes. Aging decreases the functional capacity of mitochondria through damage to mitochondrial DNA (mtDNA) over time. These damages lead to less effective oxidative phosphorylation which results in decreased ATP production and an accumulation of ROS [18,19]. This accumulation of ROS leads to increased risk of further mtDNA damage due to its proximity to the electron transport chain and lack of proactive histones unlike nuclear DNA. Furthermore, this can lead to inflammation and possibly eventual cell death. The exacerbation of mitochondrial damage and impaired function continues through the pathogenesis of AMD compounding upon the natural mtDNA mutations that come with age (Figure 1).

In addition to the lack of cellular homeostasis, the mitochondrial damage activates inflammation and apoptotic pathways due to the cells inability to produce sufficient ATP and having excessive stress from ROS. To maintain homeostasis and relieve excess ROS, there is an activation of mitochondrial permeability transition and caspase enzymes. The initial increase in mitochondrial permeability attempts to release the buildup of excessive molecules such as calcium and ROS but leads to caspase mediated apoptosis if the stress continues to overwhelm the cell [20,21]. The compounding effects of aging, oxidative stress, and this cyclic pathology also decrease the cell’s ability to remove damaged mitochondria from the RPE. The high ROS additionally damages the proteins used in autophagy and eventual mitophagy failure [22]. The damaged, dysfunctional mitochondria continue to accumulate due to failure of cellular machinery and repair systems. Excessive ROS from these damaged mitochondria continue to create a toxic cellular environment, spreading to affect other healthy mitochondria through further mtDNA damage, excessive calcium release, mitophagy machinery failure, and other factors (Figure 2).

The self-perpetuating cycle of oxidative stress and cellular damage leads to degeneration of the RPE which is a key factor in AMD progression. Thus, the critical role of mitochondria in this cycle is indicative of the connection between AMD and mitochondrial dysfunction.

It is important to note that quantifying the amount of mitochondrial dysfunction within the RPE is still limited. Current evidence is from experimental models and indirect measurements which do not directly allow us to fully understand the mitochondria within the retina due to measurements having to be done on surrogate tissues. Additionally, current literature relies upon in vitro models and animal studies which lack the full complexity of human disease [23,24]. These various methods made it difficult to compare studies and determine the extent of mitochondrial dysfunction within AMD.

### ER Downstream Stress and Its Role

The endoplasmic reticulum (ER) is fundamental for cellular functions of protein folding, calcium storage, and intracellular signaling. In the RPE this is fundamental for cellular homeostasis, repair, and clean-up processes. These functions are particularly essential in the RPE cells due to the high metabolic activity of RPE and exposure to chronic oxidative stress. The interruption of the usual functions of ER leads to misfolded or unfolded protein accumulation which creates a condition called ER stress. In response to this decreased efficiency, the ER further slows down the production of new proteins and degradation of misfolded proteins through a process called Unfolded Protein Response (UPR) (Figure 2). UPR is a protective mechanism to restore cellular homeostasis through an adaptive signaling network [25,26]. However, this mechanism may lead to damage through inflammation and apoptosis if ER stress is not relieved. Chronic activation of these pathways leads to pro-apoptotic signaling in the RPE which is a key feature of AMD progression. Additionally, inflammatory pathways via innate immune response are initiated with an increased recruitment of pro-inflammatory cytokines. These reactions cause a chronic inflammation within the retina and exacerbate the cellular damage and disease progression.

### ER and Mitochondria Associated Membranes

While studies have explored the individual pathogenic contributions of the ER and mitochondria, there is little discussion of how the two may interconnectedly contribute to the pathogenesis of AMD. The two organelles were discovered to have physical connections between them, “based on co-sedimentation of ER particles with mitochondria and electron microscopic observations of close associations between mitochondria and ER vesicles” [27–29]. These physical linkages between the two are called mitochondrial associated membranes (MAMs). These areas of direct physical linkage between the ER and mitochondria may provide a potential explanation for the disruption of homeostasis and functionality of both organelles. MAMs are discussed in playing a direct role in calcium signaling, lipid synthesis, and intracellular signaling upon the mitochondria. If impaired, this would lead to damage upon the mitochondria and conversely, the mitochondria would affect the ER at these MAM interfaces.

The ER releases calcium at the MAM sites for mitochondrial uptake which is imperative for ATP synthesis, membrane potential, and respiration. In turn, these processes cause the production of ROS. However, in times of age-related dysfunction or other causes of ER stress such as structural MAM changes, the release of calcium may increase to maintain homeostasis. This excess calcium release leads to excess ROS, leading to mitochondrial and ER damage via the MAM interface. This indicates the importance of MAMs as sites of calcium regulation and reveals how the connection can directly injure either organelle due to one’s dysfunction [30,31]. Furthermore, this excessive calcium uptake can lead to opening the mitochondrial permeability transition pore which disrupts the regulation and diminishes the proton gradient necessary for ATP production. Eventually this “leak” leads to mitochondrial swelling, outer membrane rupture, and pro-apoptotic protein release. This demise can occur rapidly in the high energy RPE cells, contributing to AMD patients’ retinal thinning and vision loss [32–34].

The ER is essential for lipid and cholesterol biosynthesis and its close contact with mitochondria is to actively regulate the biogenesis, remodeling, and function of the organelle. Dysregulation of the lipid biosynthesis pathway has been strongly implicated in retinal degeneration and specifically in AMD [35]. While the exact cellular mechanism between lipid dysregulation and downstream effects are currently unknown, studies have found lipid imbalance in damaged retinal cells, implicating a strong correlation between lipids dysregulation and AMD [36,37]. A deficiency in lipids such as cholesterol leads to weak cell membrane integrity and accumulation of these cholesterols increases inflammation and oxidative stress. Specific lipid species including 27-hydroxycholesterol and 7-ketocholesterol have been discussed as primary drivers of ER stress, activating UPR signaling in retinal cells. Ultimately, this would continue to drive the cell towards dysfunction and damage, perpetuating the cycle of damage.

MAMs play a critical role in apoptotic intracellular signaling as it mediates a cycle of oxidative stress between the mitochondria and the ER The methods discussed above of calcium signaling, UPR signaling, and lipid biosynthesis dysregulation are all collective contributing to driving increased ROS and a damaging environment for these organelles. In AMD patients, it has been shown that their RPE cells produce more ROS than normal donors, confirming this theory as a possible pathway for disease [38]. High ROS production cannot be cleared by cellular machinery due to inability to increase superoxide dismutase (SOD) [39]. The overwhelming ROS produced promotes production of hydrogen peroxide, inflammatory responses, and triggers mitochondrial mediated apoptotic signaling. In specific, the recruitment of macrophages, complement activation, and microglial activation have been documented [40–42]. Thus, MAMs are a central interface where the effects of these dysregulations compound and lead to cellular degeneration.

### Clinical Implications

Understanding the connection between the ER and mitochondria provides us with a better understanding of how AMD progresses and opens possibilities for therapeutic strategies. The understanding of multiple compound effects rather than simply mitochondrial damage suggests that targeting a single pathway through treatment may be insufficient. However, the research suggests that the accumulation of damaging effects must compound before resulting in cell death and thus, poses an opportunity for early intervention. Additionally, the various pathways to cellular degeneration may explain the heterogeneity amongst AMD patient presentations and could potentially direct us towards more personalized treatment plans.

### Potential Biomarkers for ER-Mitochondrial Dysfunction Diagnosis

The role of MAMs and the ER-Mitochondrial connected dysfunction allow us to better understand the types of damage within the body and how it may present as measurable biomarkers. In specific, Biomarkers reflecting oxidative stress, lipid dysregulation, mitochondrial injury, and ER stress signaling may provide significant clues to the presence and progression of AMD.

Oxidative stress is the predominant biomarker in AMD, especially due to the high RO species produced from cell dysfunction. Excessive ROS leads to DNA damage, lipid peroxisomes, and protein oxidation. Superoxide dismutase, oxidized lipids, and superoxide levels are correlated to imbalance within cellular systems. Biomarkers of ER level stress include UPR signaling response components, CHOP, and GRP78 (BiP) [43]. These signaling molecules amounts can provide clear knowledge of possible apoptotic signaling and increased oxidative stress. From the contribution of mitochondria to AMD pathogenesis, mitochondrial DNA damage and mitochondrial-derived peptides (MDPs) are novel biomarkers for overall mitochondrial health. MDPs are particularly important to note as their decreased levels and dysfunction are reflective of impaired mitochondrial signaling and possible degeneration. Humanin is noted to be a MDP with a cytoprotective role for RPE cells and promotes anti-apoptotic signaling [44,45]. Measurement of these MDPs reflect the stress status of the mitochondria, making it a strong candidate as a biomarker for AMD [46].

Lack of lipid homeostasis is another pathway in which biomarkers may be identified. Accumulation of oxidized lipids, cholesterol, and lipoproteins within the retina are often recognized as indicators of disease [47,48]. One study mentions ApoE as being a potential biomarker as the protein is essential for lipid transport and abnormal levels in addition to oxidized products may be indicative of retinal diseases. The accumulation of dysregulated lipids in AMD may present as lipid deposits (known as drusen) behind the RPE, contributing to chronic inflammation, inhibition of nutrient and oxygen exchange, and increased oxidative stress. Drusen are early signs of AMD and contain ApoE, allowing for a better understanding of disease progression and presence [49].

MAM-related biomarkers have been investigated in association to type 2 diabetes mellitus, However, this finding can be useful for AMD diagnosis due to overlapping pathophysiology in the retina. It was noted that MAM dysfunction can trigger systemic responses detectable in peripheral blood transcriptomics. Researchers discovered three genes upregulated in type 2 diabetes mellitus diseased individuals and identified their mRNA within red blood cells as possible biomarkers. A similar procedure can be applied to AMD, leaving room for a novel biomarker and method of collection.

### Possible Treatments for AMD along the ER-Mitochondrial Axis

Currently, treatments focus upon decreasing oxidative stress, cellular damage, and mitigating symptoms. MAMs, the ER, and the mitochondria have not been used as targeted therapies but have been identified as possibilities for future drug developments.

Antioxidant therapies have been under investigation in hopes of decreasing the amount of ROS produced and to ultimately prevent damage to RPE cells. A randomized clinical trial found that combinations of Vitamin C, Vitamin E, beta carotene, and zinc oxide delay the progression of AMD. It was noted that this combination may be a beneficial secondary prevention in managing patients’ symptoms and progression [50].

Inhibiting the complement system and preventing further exacerbation of cellular inflammation and apoptosis is another pathway utilized to slow AMD progression. In particular, FDA approved drugs like Izervay (avacincaptad pegol) and Pegcetacoplan (SYFOVRE) which are intravitreal injections. The inhibition along the complement cascade prevents further damage to RPE cells and ultimately slows disease progression [51,52].

Tissue engineering and RPE cell transplantation have also been considered as treatment options but are limited by ethical, financial, and immunological issues [53,54].

The limited therapeutic options for AMD stems from the complexity and multifactorial nature of the disease. However, the discovery of MAMs and understanding of the pathophysiology of AMD opens the door for future breakthroughs.

## Figures and Tables

**Figure 1:**
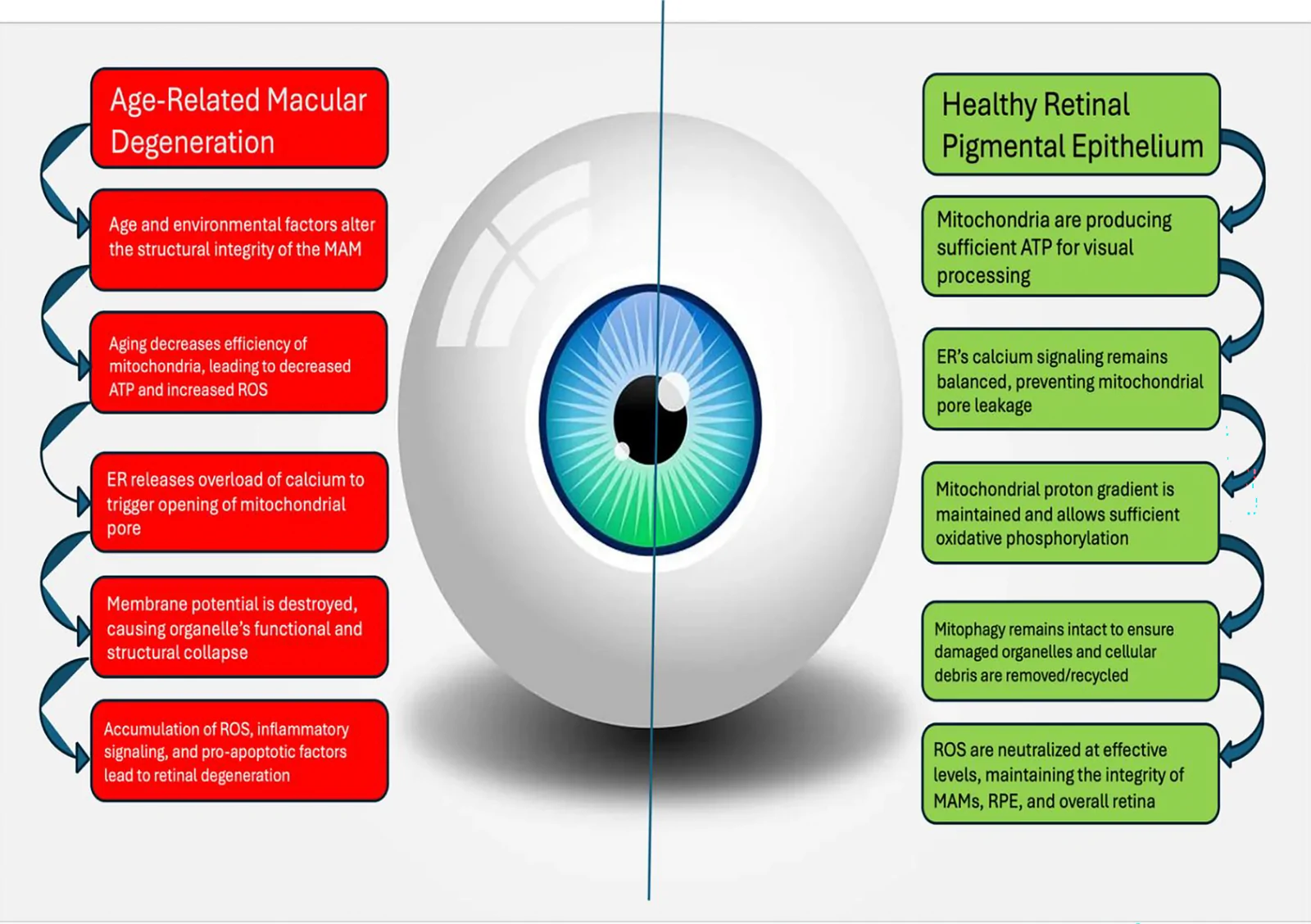
Pathological mechanism of ER-mitochondrial dysfunction in the retinal pigment epithelium (RPE) of AMD versus healthy subjects. The comparative figure provides a breakdown of the steps leading to retinal degeneration on the left, while the functional necessities for maintaining a healthy retina are detailed on the right.

**Figure 2:**
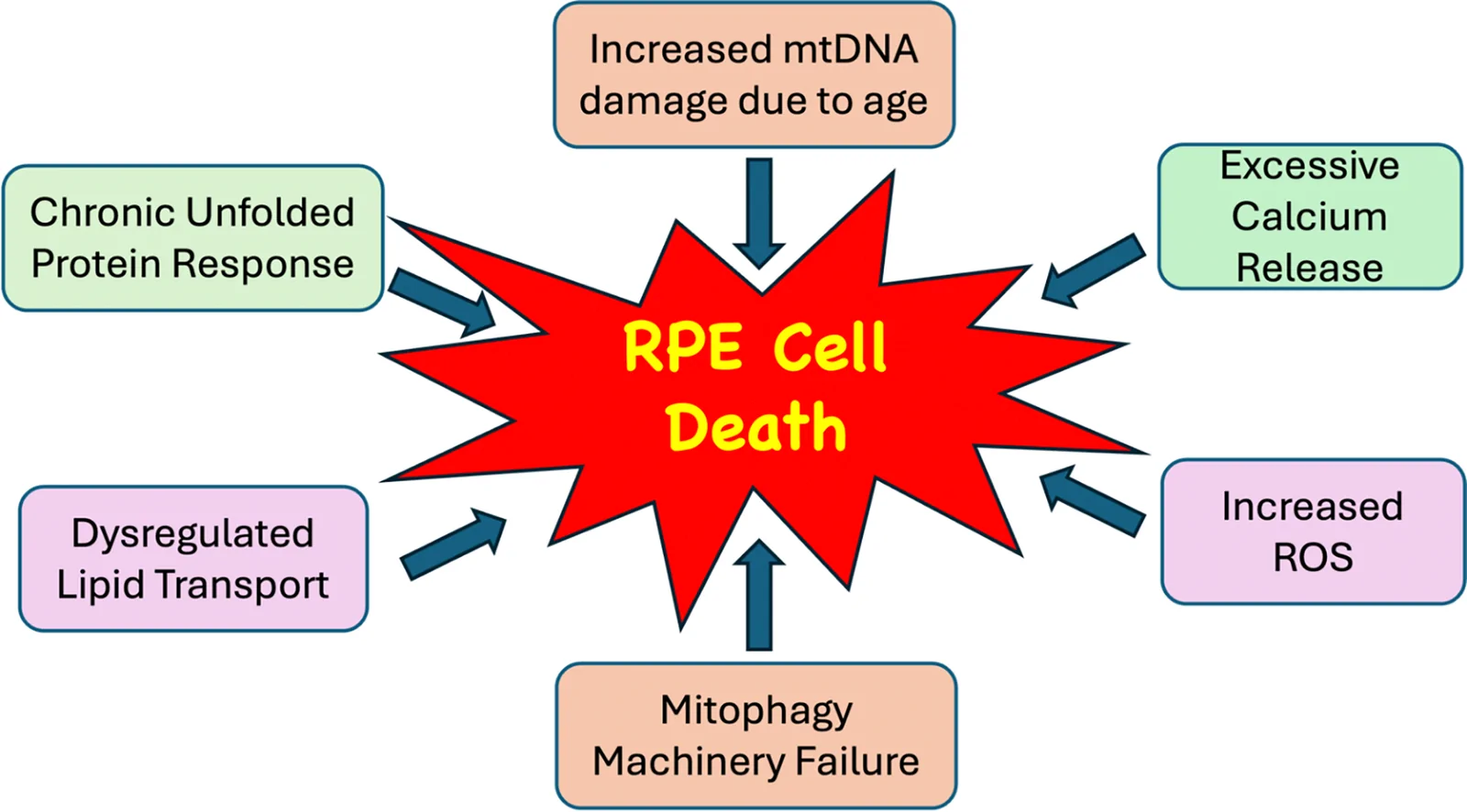
Multifactorial intracellular factors contributing to the cell death of retinal pigment epithelium (RPE) in Age-Related Macular Degeneration. This figure provides a visual of the various pathways that converge to result in the characteristic cellular damage in AMD.

## Data Availability

No new pre-clinical or clinical studies were performed, and all findings were gathered from the published articles and critically reviewed.

## Key Points

- Aging, smoking, light exposure, and other epigenetic factors are chronic oxidative stressors.
- Mitochondrial associated membranes (MAMs) are the physical and functional bridge between the mitochondria and endoplasmic reticulum (ER) andt critically regulate essential cellular functions including calcium signaling, mitochondrial dynamics, and apoptosis.
- Chronic oxidative stressors cause early mitochondrial DNA (mtDNA) damage and electron transport chain breakdown in the Retinal Pigment Epithelium (RPE), resulting in the failure of MAM communication triggering ATP crisis and progressive tissue deterioration.
- Dysfunction in the mitochondria-ER interaction may cease functional protein folding in the ER, prompting to maladaptive ER stress, leading to progressive retinal degeneration through the production of reactive oxygen species (ROS), calcium overload, and inflammasome activation in RPE cells.
- Calcium overload due to dysregulated transfer from ER to mitochondria results in the hyperactivation of the NLRP3 inflammasomes, mitochondrial matrix swelling, and cytochrome c leakage.
- Unfolded protein response (UPR) due to high reactive oxygen species (ROS) disrupts redox balance in the ER lumen and activates ER stress transducers leading to the activation of apoptotic cascade.
- Dysregulated lipid and cholesterol transport due to blockade or aberrant lipid transfer between the ER and mitochondrial membranes results in the accumulation of cholesterol in mitochondria driving electron leakage and lipid peroxidation (ferroptosis).
- Stabilization of mitochondrial associated membranes (MAMs) architecture or inhibiting downstream effectors could be an effective strategy to treat dry age-related macular degeneration (AMD).
- Use of the targeted antioxidants and mitochondrial protective agents, inhibitors of complement system, and the strategy to activate master transcriptional regulators, such as Nrf2 and PGC-1α pathways, could be promising therapy to restore antioxidant defenses and to improve ER proteostasis and mitochondrial biogenesis and thus preventing RPE disintegration.

## References

[1] Singh RB, Stettler I, Romano F, Prevalence of Age-Related Macular Degeneration in the United States: A Medicare-Based Analysis from 2014 to 2021. Ophthalmic Res 68 (2025): 573–582. 10.1159/000548724 PMC12705084 41194565

[2] Qu S, Lin H, Pfeiffer N, Age-Related Macular Degeneration and Mitochondria-Associated Autoantibodies: A Review of the Specific Pathogenesis and Therapeutic Strategies. Int J Mol Sci 25 (2024): 1624. 10.3390/ijms25031624 PMC10855900 38338904

[3] Tokarz P, Kaarniranta K, Blasiak J. Role of Antioxidant Enzymes and Small Molecular Weight Antioxidants in the Pathogenesis of Age-Related Macular Degeneration (AMD). Biogerontology 14 (2013): 461–482. 10.1007/s10522-013-9463-2 PMC3824279 24057278

[4] Somasundaran S, Constable IJ, Mellough CB, Retinal Pigment Epithelium and Age-Related Macular Degeneration: A Review of Major Disease Mechanisms. Clin Exp Ophthalmol 48 (2020): 1043–1056. 10.1111/ceo.13834 PMC7754492 32710488

[5] Yang S, Zhou J, Li D. Functions and Diseases of the Retinal Pigment Epithelium. Front Pharmacol 12 (2021): 727870. 10.3389/fphar.2021.727870 PMC8355697 34393803

[6] Allen RT, Cluck MW, Agrawal DK. Mechanisms Controlling Cellular Suicide: Role of Bcl-2 and Caspases. Cell Mol Life Sci 54 (1998): 427–445. 10.1007/s000180050171 PMC11147391 9645223

[7] Mari YM, Fraix MP, Agrawal DK. Pulmonary Fibrosis and Diabetes Mellitus: Two Coins with the Same Face. Arch Intern Med Res 7 (2024): 53–70. 10.26502/aimr.0165 PMC10994216 38576768

[8] Chaudhary F, Agrawal DK. Anesthesia-Induced Developmental Neurotoxicity in Pediatric Population. J Surg Res (Houst) 7 (2024): 490–500. 10.26502/jsr.10020400 PMC11649317 39687550

[9] Khwaja B, Thankam FG, Agrawal DK. Mitochondrial DAMPs and Altered Mitochondrial Dynamics in OxLDL Burden in Atherosclerosis. Mol Cell Biochem 476 (2021): 1915–1928. 10.1007/s11010-021-04061-0 33492610

[10] Brown SM, Larsen NK, Thankam FG, Fetal Cardiomyocyte Phenotype, Ketone Body Metabolism, and Mitochondrial Dysfunction in the Pathology of Atrial Fibrillation. Mol Cell Biochem 476 (2021): 1165–1178. 10.1007/s11010-020-03980-8 PMC7878362 33188453

[11] Thankam FG, Ayoub JG, Ahmed MMR, Association of Hypoxia and Mitochondrial Damage Associated Molecular Patterns in the Pathogenesis of Vein Graft Failure: A Pilot Study. Transl Res 229 (2021): 38–52. 10.1016/j.trsl.2020.08.010 PMC7867581 32861831

[12] Thankam FG, Chandra IS, Kovilam AN, Amplification of Mitochondrial Activity in the Healing Response Following Rotator Cuff Tendon Injury. Sci Rep 8 (2018): 17027. 10.1038/s41598-018-35391-7 PMC6242817 30451947

[13] Kumar V Endoplasmic Reticulum-Mitochondrial Crosstalk in Neurodegenerative and Eye Diseases. Neurology (ECronicon) 11 (2019): 864–873. PMC6746603 31528859

[14] Malicevic U, Skrbic R, Agrawal DK. Metabolically Induced Intestinal Inflammation: The Role of ER Stress and Autophagy in a Porcine Model of Diabetes. Mol Cell Biochem (2026). 10.1007/s11010-026-05659-y PMC13638702 42484790

[15] Malicevic U, Rai V, Skrbic R, Intricate Interplay Between ORMDL3, ER Stress, and Autophagy in the Diabetic Intestine. Mol Cell Biochem 481 (2026): 791–807. 10.1007/s11010-025-05424-7 PMC12963212 41182648

[16] Kaarniranta K, Uusitalo H, Blasiak J, Mechanisms of Mitochondrial Dysfunction and Their Impact on Age-Related Macular Degeneration. Prog Retin Eye Res 79 (2020): 100858. 10.1016/j.preteyeres.2020.100858 PMC7650008 32298788

[17] Cano M, Wang L, Wan J, Oxidative Stress Induces Mitochondrial Dysfunction and a Protective Unfolded Protein Response in RPE Cells. Free Radic Biol Med 69 (2014): 1–14. 10.1016/j.freeradbiomed.2014.01.004 PMC3960355 24434119

[18] Lenin RR, Koh YH, Zhang Z, Dysfunctional Autophagy, Proteostasis, and Mitochondria as a Prelude to Age-Related Macular Degeneration. Int J Mol Sci 24 (2023): 8763. 10.3390/ijms24108763 PMC10217992 37240109

[19] Jiang F, Ma J, Lei C, Age-Related Macular Degeneration: Cellular and Molecular Signaling Mechanisms. Int J Mol Sci 26 (2025): 6174. 10.3390/ijms26136174 PMC12249930 40649951

[20] Kaarniranta K, Blasiak J, Liton P, Autophagy in Age-Related Macular Degeneration. Autophagy 19 (2023): 388–400. 10.1080/15548627.2022.2069437 PMC9851256 35468037

[21] Sridevi Gurubaran I, Viiri J, Koskela A, Mitophagy in the Retinal Pigment Epithelium of Dry Age-Related Macular Degeneration Investigated in the NFE2L2/PGC-1α−/− Mouse Model. Int J Mol Sci 21 (2020): 1976. 10.3390/ijms21061976 PMC7139489 32183173

[22] Saneto RP, Perez FA. Mitochondria-Associated Membrane Scaffolding with Endoplasmic Reticulum: A Dynamic Pathway of Developmental Disease. Front Mol Biosci 9 (2022): 908721. 10.3389/fmolb.2022.908721 PMC9237565 35775081

[23] Klemmensen MM, Borrowman SH, Pearce C, Mitochondrial Dysfunction in Neurodegenerative Disorders. Neurotherapeutics 21 (2024): e00292. 10.1016/j.neurot.2023.10.002 PMC10903104 38241161

[24] Ebeling MC, Polanco JR, Qu J, Improving Retinal Mitochondrial Function as a Treatment for Age-Related Macular Degeneration. Redox Biol 34 (2020): 101552. 10.1016/j.redox.2020.101552 PMC7327959 32446621

[25] Salminen A, Kauppinen A, Hyttinen JM, Endoplasmic Reticulum Stress in Age-Related Macular Degeneration: Trigger for Neovascularization. Mol Med 16 (2010): 535–542. 10.2119/molmed.2010.00070 PMC2972399 20683548

[26] Carreras-Sureda A, Pihán P, Hetz C. The Unfolded Protein Response: At the Intersection Between Endoplasmic Reticulum Function and Mitochondrial Bioenergetics. Front Oncol 7 (2017): 55. 10.3389/fonc.2017.00055 PMC5377016 28421160

[27] Mannella CA, Buttle K, Rath BK, Electron Microscopic Tomography of Rat-Liver Mitochondria and Their Interaction with the Endoplasmic Reticulum. Biofactors 8 (1998): 225–228. 10.1002/biof.5520080309 9914823

[28] Meier PJ, Spycher MA, Meyer UA. Isolation and Characterization of Rough Endoplasmic Reticulum Associated with Mitochondria from Normal Rat Liver. Biochim Biophys Acta 646 (1981): 283–297. 10.1016/0005-2736(81)90335-7 6170330

[29] Shore GC, Tata JR. Two Fractions of Rough Endoplasmic Reticulum from Rat Liver. I. Recovery of Rapidly Sedimenting Endoplasmic Reticulum in Association with Mitochondria. J Cell Biol 72 (1977): 714–725. 10.1083/jcb.72.3.714 PMC2111030 838772

[30] Marchi S, Patergnani S, Missiroli S, Mitochondrial and Endoplasmic Reticulum Calcium Homeostasis and Cell Death. Cell Calcium 69 (2018): 62–72. 10.1016/j.ceca.2017.05.003 28515000

[31] Janikiewicz J, Szymanski J, Malinska D, Mitochondria-Associated Membranes in Aging and Senescence: Structure, Function, and Dynamics. Cell Death Dis 9 (2018): 332. 10.1038/s41419-017-0105-5 PMC5832430 29491385

[32] Kasi A, Steidl W, Kumar V. Endoplasmic Reticulum-Mitochondria Crosstalk in Fuchs Endothelial Corneal Dystrophy: Current Status and Future Prospects. Int J Mol Sci 26 (2025): 894. 10.3390/ijms26030894 PMC11817211 39940664

[33] Paillusson S, Stoica R, Gomez-Suaga P, There’s Something Wrong with My MAM: The ER-Mitochondria Axis and Neurodegenerative Diseases. Trends Neurosci 39 (2016): 146–157. 10.1016/j.tins.2016.01.008 PMC4780428 26899735

[34] Kaštelan S, Antunica AG, Konjevoda S, Mitochondrial ROS in Retinal Neurodegeneration: Thresholds, Quality Control Failure, and Precision Therapeutic Windows. Biomolecules 16 (2026): 445. 10.3390/biom16030445 PMC13024072 41897380

[35] Zhang SX, Wang JJ, Starr CR, The Endoplasmic Reticulum: Homeostasis and Crosstalk in Retinal Health and Disease. Prog Retin Eye Res 98 (2024): 101231. 10.1016/j.preteyeres.2023.101231 PMC11056313 38092262

[36] Molday RS, Zhang K. Defective Lipid Transport and Biosynthesis in Recessive and Dominant Stargardt Macular Degeneration. Prog Lipid Res 49 (2010): 476–492. 10.1016/j.plipres.2010.07.002 PMC2946192 20633576

[37] Zhou S, Taskintuna K, Hum J, PGC-1α Repression Dysregulates Lipid Metabolism and Induces Lipid Droplet Accumulation in Retinal Pigment Epithelium. Cell Death Dis 15 (2024): 385. 10.1038/s41419-024-06762-y PMC11144268 38824126

[38] Hyttinen JMT, Viiri J, Kaarniranta K, Mitochondrial Quality Control in AMD: Does Mitophagy Play a Pivotal Role? Cell Mol Life Sci 75 (2018): 2991–3008. 10.1007/s00018-018-2843-7 PMC11105454 29777261

[39] McCord JM, Edeas MA. SOD, Oxidative Stress and Human Pathologies: A Brief History and a Future Vision. Biomed Pharmacother 59 (2005): 139–142. 10.1016/j.biopha.2005.03.005 15862706

[40] Kinnunen K, Petrovski G, Moe MC, Molecular Mechanisms of Retinal Pigment Epithelium Damage and Development of Age-Related Macular Degeneration. Acta Ophthalmol 90 (2012): 299–309. 10.1111/j.1755-3768.2011.02179.x 22112056

[41] Mettu PS, Wielgus AR, Ong SS, Retinal Pigment Epithelium Response to Oxidant Injury in the Pathogenesis of Early Age-Related Macular Degeneration. Mol Aspects Med 33 (2012): 376–398. 10.1016/j.mam.2012.04.006 22575354

[42] Tuo J, Grob S, Zhang K, Genetics of Immunological and Inflammatory Components in Age-Related Macular Degeneration. Ocul Immunol Inflamm 20 (2012): 27–36. 10.3109/09273948.2011.628432 PMC3292785 22324898

[43] Bilbao-Malavé V, González-Zamora J, de la Puente M, Mitochondrial Dysfunction and Endoplasmic Reticulum Stress in Age-Related Macular Degeneration, Role in Pathophysiology, and Possible New Therapeutic Strategies. Antioxidants (Basel) 10 (2021): 1170. 10.3390/antiox10081170 PMC8388889 34439418

[44] Sreekumar PG, Hinton DR, Kannan R. Endoplasmic Reticulum-Mitochondrial Crosstalk: A Novel Role for the Mitochondrial Peptide Humanin. Neural Regen Res 12 (2017): 35–38. 10.4103/1673-5374.198970 PMC5319229 28250736

[45] Minasyan L, Sreekumar PG, Hinton DR, Protective Mechanisms of the Mitochondrial-Derived Peptide Humanin in Oxidative and Endoplasmic Reticulum Stress in RPE Cells. Oxid Med Cell Longev (2017): 1675230. 10.1155/2017/1675230 PMC5549471 28814984

[46] Nashine S, Kenney MC. Effects of Mitochondrial-Derived Peptides (MDPs) on Mitochondrial and Cellular Health in AMD. Cells 9 (2020): 1102. 10.3390/cells9051102 PMC7290668 32365540

[47] Álvarez-Barrios A, Álvarez L, Sáenz de Santa María P, Dysregulated Lipid Metabolism in a Retinal Pigment Epithelial Cell Model and Serum of Patients with Age-Related Macular Degeneration. BMC Biol 23 (2025): 96. 10.1186/s12915-025-02198-8 PMC11993946 40221802

[48] Zhang X, Qiu B, Gong Z, Differentially Regulated Apolipoproteins and Lipid Profiles as Novel Biomarkers for Polypoidal Choroidal Vasculopathy and Neovascular Age-Related Macular Degeneration. Front Endocrinol (Lausanne) 13 (2022): 946327. 10.3389/fendo.2022.946327 PMC9343613 35928899

[49] Ana RD, Gliszczynska A, Sanchez-Lopez E, Precision Medicines for Retinal Lipid Metabolism-Related Pathologies. J Pers Med 13 (2023): 635. 10.3390/jpm13040635 PMC10145959 37109021

[50] Age-Related Eye Disease Study Research Group. A Randomized, Placebo-Controlled, Clinical Trial of High-Dose Supplementation with Vitamins C and E, Beta Carotene, and Zinc for Age-Related Macular Degeneration and Vision Loss: AREDS Report No. 8. Arch Ophthalmol 119 (2001): 1417–1436. 10.1001/archopht.119.10.1417 PMC1462955 11594942

[51] Nadeem A, Malik IA, Shariq F, Advancements in the Treatment of Geographic Atrophy: Focus on Pegcetacoplan in Age-Related Macular Degeneration. Ann Med Surg (Lond) 85 (2023): 6067–6077. 10.1097/MS9.0000000000001466 PMC10718344 38098608

[52] Shakeel L, Khan A, Akilimali A. Izervay (Avacincaptad Pegol): Paving the Way for Vision Preservation in Geographic Atrophy. Ann Med Surg (Lond) (2024). 10.1097/MS9.0000000000002021 PMC11060312 38694318

[53] Holz FG, Schmitz-Valckenberg S, Fleckenstein M. Recent Developments in the Treatment of Age-Related Macular Degeneration. J Clin Invest 124 (2014): 1430–1438. 10.1172/JCI71029 PMC3973093 24691477

[54] Coleman HR, Chan CC, Ferris FL, Age-Related Macular Degeneration. Lancet 372 (2008): 1835–1845. 10.1016/S0140-6736(08)61759-6 PMC2603424 19027484

